# A multiscale geospatial analysis integrating explainable machine learning to characterize spatial disparities in melanoma mortality associated with environmental justice and healthcare access in the contiguous United States

**DOI:** 10.3389/fpubh.2026.1866117

**Published:** 2026-08-20

**Authors:** Hongwen Song, Caijie Tian, Xiaolei Ye, Qian Li, Jun Gan, Junfeng Zheng, Dong Miao

**Affiliations:** 1 Department of Dermatology, The 989th Hospital of the People's Liberation Army Joint Logistics Support Force, Luoyang, Henan, China; 2 School Clinic, Shanghai Construction Management Vocational College, Shanghai, China; 3 Department of Infectious Disease Prevention and Control, The Center for Disease Prevention and Control in Western Theater Command of the People's Liberation Army Joint Logistics Support Force, Lanzhou, Gansu, China; 4 Department of Emergency, The 980th Hospital of the People's Liberation Army Joint Logistics Support Force (Primary Bethune International Peace Hospital of PLA), Shijiazhuang, China

**Keywords:** environmental justice, explainable artificial intelligence (XAI), melanoma mortality, multiscale geographically weighted regression (MGWR), spatial epidemiology

## Abstract

**Background:**

Melanoma mortality exhibits profound geographical disparities associated with complex sociodemographic, environmental, and healthcare factors. Traditional spatial models may not fully capture the multi-scale heterogeneity underlying these geographic disparities. This study aimed to characterize the spatial heterogeneity and operational scales of factors associated with county-level melanoma mortality across the United States.

**Methods:**

We conducted a county-level ecological study across the contiguous US (2018–2024). To address mortality data suppression by the Centers for Disease Control and Prevention (CDC) (deaths < 10), a representativeness analysis was performed. Predefined core covariates (median age, UV exposure, dermatologist density) were integrated with 45 Environmental Justice Index (EJI) indicators. Feature selection was performed using an XGBoost-SHAP pipeline followed by stepwise AICc optimization. Multiscale Geographically Weighted Regression (MGWR) was employed to evaluate spatial non-stationarity and operational scales, comparing its performance against ordinary least squares (OLS) and standard geographically weighted regression (GWR) models.

**Results:**

The analytical sample comprised 1,156 counties, representing 88.6% of the total US population. MGWR demonstrated superior explanatory power (*R*^2^ = 0.666, AICc = 2247.84) and substantially reduced residual spatial autocorrelation. The XGBoost-SHAP framework identified eight core predictors. MGWR revealed substantial variation in spatial scales: baseline spatial variation operated locally, while median age demonstrated a regional-scale positive association, with coefficients varying across geographic regions. High-volume road proximity demonstrated regional inverse associations. Dermatologist density, ozone exposure, and tornado frequency operated at global scales; dermatologist density and ozone exposure showed widespread inverse associations (potentially reflecting broader metropolitan healthcare and socioeconomic patterns), whereas tornado frequency was positively associated with melanoma mortality. Notably, UV exposure, hurricane frequency, and mobile home proportions exhibited global-scale coefficient patterns but lacked statistically significant local effects (*P* ≥ 0.05).

**Conclusions:**

Melanoma mortality exhibits multiscale spatial patterns. The limited association observed for environmental UV exposure suggests that demographic structure and healthcare-related factors may play important roles in explaining county-level mortality disparities. These findings support spatially targeted public health strategies, including improved dermatological care access in aging and disaster-prone regions.

## 1 Introduction

Malignant melanoma remains the most lethal form of skin cancer in the United States. Despite recent breakthroughs in targeted therapies and immunotherapies that have improved overall survival, the burden of melanoma mortality is profoundly unevenly distributed across geographic regions and demographic groups (1). Recent epidemiological trends indicate that while age-adjusted mortality rates are declining nationally, profound spatial disparities persist at the state and county levels, reflecting complex interactions among demographic, environmental, and healthcare-related factors beyond biological aging and UV exposure (2). Rural residents, socioeconomically disadvantaged populations, and those residing in medically underserved areas consistently exhibit more advanced disease stages at presentation and higher melanoma-specific mortality (3). Crucially, the unequal distribution of specialized healthcare resources, such as the severe maldistribution of dermatologists across US counties, has been identified as a critical systemic factor associated with these spatial inequities (4).

Beyond healthcare access, the broader paradigm of Social Determinants of Health (SDoH) has increasingly recognized the cumulative associations between environmental and socio-demographic deprivation on cancer outcomes (5). In response to the need for a comprehensive geospatial framework, the CDC recently launched the EJI. This national, place-based tool uniquely quantifies the cumulative impacts of environmental burdens (e.g., air pollution, built environment), social vulnerability, and climate-driven hazards on human health (6). While the EJI has been successfully utilized to map disparities in chronic diseases and respiratory conditions, its application in understanding spatial variations in cutaneous melanoma mortality remains largely unexplored, leaving a significant gap in oncological health geography.

High-dimensional datasets like the EJI frequently suffer from severe multicollinearity, which undermines traditional regression analyses. To address this, contemporary epidemiological research has increasingly adopted explainable artificial intelligence (XAI) frameworks, specifically Extreme Gradient Boosting (XGBoost) combined with SHapley Additive exPlanations (SHAP) (7). This methodology facilitates the characterization of complex, non-linear associations and enables data-driven feature selection without relying on rigid distributional assumptions (8, 9).

Furthermore, spatial epidemiology often utilizes GWR to capture spatial heterogeneity. However, standard GWR imposes a restrictive assumption by limiting all covariates to a single spatial scale (bandwidth) (10). MGWR overcomes this limitation by optimizing a unique spatial bandwidth for each predictor, thereby allowing estimation of the geographic scales at which associations between environmental, social, and healthcare-related factors vary (11, 12).

To bridge existing methodological and geographical gaps, this study proposes an analytical framework integrating machine learning and spatial econometrics to evaluate factors associated with county-level melanoma mortality in the contiguous United States (2018–2024). After validating the sample's representativeness, we utilize an XGBoost-SHAP pipeline to distill complex, non-linear relationships and identify the most important predictors from the EJI alongside established environmental and sociodemographic covariates. Moving beyond global regression estimations, we subsequently deploy the optimal MGWR model. This approach enables us to characterize spatial heterogeneity and unique operational scales of the identified associations, offering a robust foundation for addressing environmental injustice in skin cancer outcomes.

## 2 Materials and methods

### 2.1 Study area and spatial data linkage

An ecological, county-level, cross-sectional study was conducted across the United States. To ensure spatial contiguity and model robustness, the analytical sample was determined through a strict three-step selection process: (1) location within the contiguous United States, excluding Hawaii, Alaska, and US territories due to documented inconsistencies in environmental data availability (13); (2) availability of unsuppressed melanoma mortality records in the CDC WONDER database, which necessitated the exclusion of sparsely populated rural counties with fewer than 10 deaths due to federal privacy protocols; and (3) complete data across all predefined covariates and EJI indicators. Spatial boundary files for contiguous counties were obtained from the US Census Bureau's TIGER/Line Shapefiles. To construct the spatial database, demographic, environmental, and healthcare datasets were aggregated and linked using the unique 5-digit Federal Information Processing Standard (FIPS) codes (14).

Recognizing that data suppression by federal health agencies primarily affects sparsely populated rural counties, a representativeness analysis was performed. We compared the sociodemographic characteristics of the included analytical sample vs. the excluded counties using the two-sided Mann–Whitney *U* test, thereby ensuring the external validity of our spatial framework.

### 2.2 Dependent variable: melanoma mortality

Melanoma mortality data spanning a 7-year window (2018–2024) were extracted from the CDC WONDER Multiple Cause of Death database. Cases were identified using the International Classification of Diseases, Tenth Revision (ICD-10) code C43 (Malignant melanoma of skin) as the underlying cause of death.

Under the US Health Insurance Portability and Accountability Act (HIPAA) privacy protocols, CDC WONDER suppresses death counts fewer than 10 at the county level. Furthermore, age-adjusted rates cannot be requested simultaneously with county-level geographical queries to prevent deductive disclosure. To overcome this systemic limitation, the dependent variable was formulated as the average annual crude mortality rate (per 100,000 residents). To rigorously mitigate the potential demographic confounding inherent to crude rates in spatial epidemiology, the county-level median age was integrated into the regression framework as a predefined core covariate, effectively controlling for population aging without violating suppression rules (15).

### 2.3 Independent variables and predefined covariates

We synthesized a high-dimensional dataset capturing diverse environmental and social exposures, and predefined three variables as core covariates: county-level median age, dermatologist density, and environmental UV exposure. Specifically, the population age profile was represented by the median age of the county population in 2020, obtained from the Health Resources and Services Administration (HRSA) Area Health Resources Files (AHRF). While proportion-based age metrics, such as the percentage of residents aged 65 or older, are widely studied in dermatological epidemiology (16, 17), absolute median age was selected to capture the central tendency of the demographic structure as a continuous baseline covariate. Healthcare access was represented by dermatologist density, calculated as the number of resident non-federal dermatologists per 100,000 population in 2023, also derived from the HRSA AHRF. Lastly, environmental carcinogen exposure was defined as the daily mean ultraviolet irradiance (Wh/m^2^) averaged from 2020 to 2024, acquired from the National Cancer Institute (NCI).

To evaluate multi-dimensional environmental and social burdens, we utilized the EJI 2024, developed by the CDC and the Agency for Toxic Substances and Disease Registry (ATSDR) (18). We extracted detailed indicators (ranging from 0 to 1 as percentile ranks) encompassing social vulnerability, environmental burden, and climate vulnerability. Tract-level EJI metrics were aggregated to the county level via population-weighted averaging. A comprehensive data dictionary is provided in Supplementary Table S1.

### 2.4 Machine learning-based feature selection

To reduce multicollinearity and minimize redundancy among the EJI indicators, a rigorous dimensionality reduction pipeline was implemented.

First, multicollinearity was diagnosed using the Variance Inflation Factor (VIF). An iterative exclusion algorithm was employed, sequentially removing the indicator with the highest VIF until all remaining variables achieved a VIF < 10 (19).

Second, the retained variables underwent non-linear importance ranking using the Extreme Gradient Boosting (XGBoost) algorithm (20). To evaluate the robustness of the feature importance rankings, a five-fold cross-validation procedure was performed during model development. The global contribution of each feature was then quantified using the mean absolute SHapley Additive exPlanations (SHAP) value, an explainable artificial intelligence (XAI) approach that robustly captures complex variable interactions (21).

Because multiscale geographically weighted regression assumes additive linear relationships, variables identified solely through tree-based non-linear importance may not necessarily provide meaningful linear contributions. To reconcile the non-linear feature selection process with the linear spatial regression framework, we adopted a two-stage funnel approach. Candidates for inclusion were derived from the top ten highest ranked variables in the cross validated SHAP analysis and were sequentially introduced to the base model. A variable was retained only if its inclusion yielded a reduction in the Corrected Akaike Information Criterion of greater than 2.0 (22). This procedure ensured that only variables providing meaningful additive linear contributions were retained in the final MGWR model.

### 2.5 Multiscale geographically weighted regression (MGWR)

Traditional Global OLS regression assumes spatial stationarity. To account for spatial heterogeneity, geographically weighted regression (GWR) is commonly utilized. However, standard GWR restricts all covariates to a single spatial bandwidth, which contradicts the geographical reality that distinct processes operate at different spatial scales (22).

Therefore, we adopted the MGWR model. MGWR relaxes the assumption of a universal spatial scale by optimizing a specific bandwidth for each predictor (23). The MGWR model is expressed as:


$$\begin{array}{l} Y_{i}=\beta_{0\left(bw_{0}\right)}\left(u_{i},v_{i}\right)+\overset{p}{\underset{k=1}{\sum }}\beta_{k\left(bw_{k}\right)}\left(u_{i},v_{i}\right)X_{ik}+\varepsilon_{i} \end{array}$$ (1)

where *Y*_*i*_ is the standardized crude mortality rate at county *i*; (*u*_*i*_, *v*_*i*_) denotes the geographic centroid coordinates; β_0(*b*_*w*__0_)_ is the local intercept with its specific bandwidth *bw*_0_; β_*k*(*b*_*w*__*k*_)_ represents the local coefficient of the *k*-th variable with its unique bandwidth *bw*_*k*_; and ε_*i*_ is the error term.

All variables were *Z*-score standardized prior to modeling. The spatial kernel was calibrated using an adaptive bi-square function, and optimal bandwidths were determined via a back-fitting algorithm optimized by the AICc (24).

#### 2.5.1 Spatial diagnostics and scale interpretation

To evaluate the spatial autocorrelation of model residuals, Moran's *I* was computed. Given the discontinuous nature of our spatial map, a *K*-Nearest Neighbors (KNN, *K* = 8) spatial weight matrix was utilized instead of traditional contiguity weights to bridge geographical gaps.

The spatial scale of each predictor was subsequently categorized based on its optimal bandwidth relative to the total number of observations (*N* = 1,156). Specifically, variables with bandwidths representing less than 10% of N were classified as operating at a local scale, indicating high spatial heterogeneity. Bandwidths encompassing between 10% and 75% of N were designated as regional scale, reflecting broader geographical gradients. Finally, bandwidths exceeding 75% of N were defined as global scale, reflecting spatial stationarity where the predictor's marginal impact remains uniform nationwide (23).

To evaluate whether the observed protective associations of high-volume road proximity and ozone exposure were attributable to underlying urbanicity rather than direct environmental effects, a sensitivity analysis was conducted by incorporating county-level urban-rural classification as an additional covariate. Urbanicity was defined using the 2023 USDA Rural–Urban Continuum Codes (RUCC), with metropolitan counties (RUCC codes 1–3) classified as urban counties and non-metropolitan counties (RUCC codes 4–9) classified as rural counties. The MGWR model was refitted after adjustment for this binary urbanicity indicator, and the changes in coefficients, statistical significance, and direction consistency for EPL_ROAD and EPL_OZONE were evaluated relative to the original model.

### 2.6 Statistical analysis

All statistical tests were two-sided, and a *P*-value < 0.05 was considered statistically significant. Descriptive statistics for continuous variables are expressed as medians with interquartile ranges (IQR). Differences between the included analytical sample and excluded counties were assessed using the non-parametric Mann–Whitney *U* test. Data preprocessing and machine learning were conducted using Python 3.10 (Python Software Foundation, Wilmington, DE, USA). Spatial regression modeling was performed using the mgwr package (version 2.1.2) (24). Spatial mapping was conducted using ArcGIS 10.8 (ESRI Inc., Redlands, CA, USA).

## 3 Results

### 3.1 Sample characteristics and representativeness

**Figure 1:**
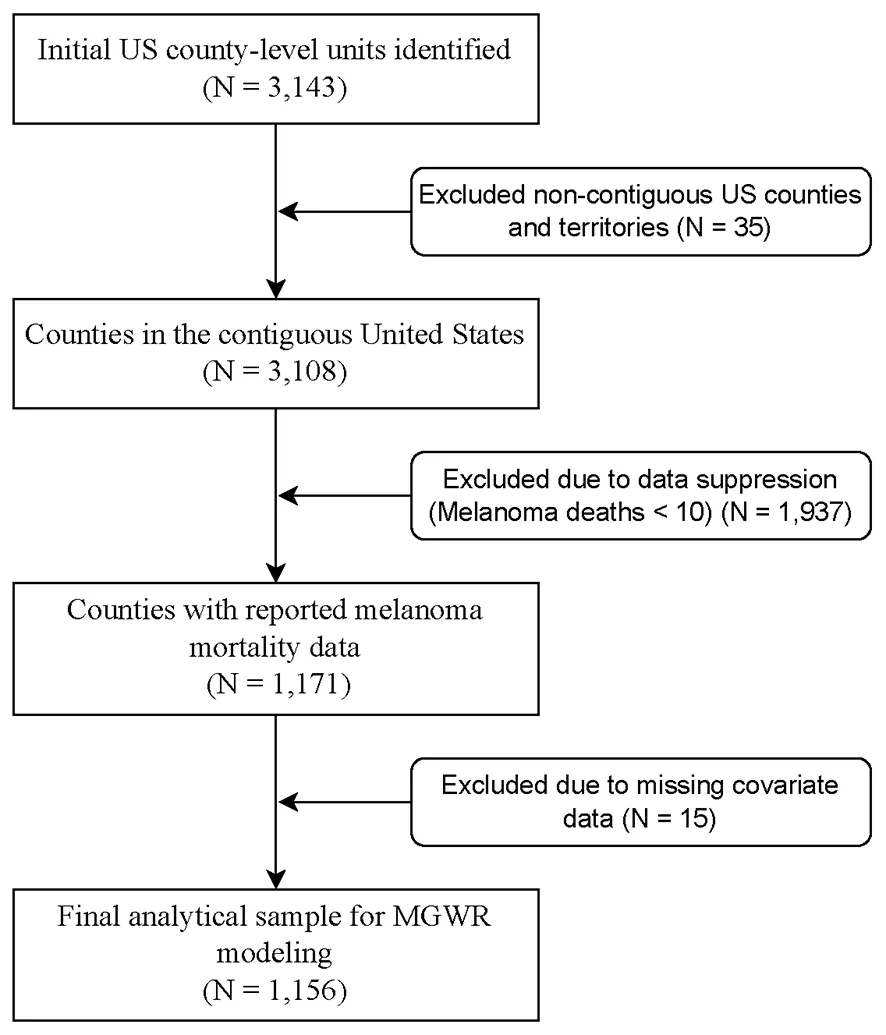
Flowchart of the sample selection process for analyzing county-level melanoma mortality across the contiguous United States (2018–2024).

The spatial sample selection process is detailed in Figure 1. Of the initial 3,108 counties in the contiguous United States, a total of 1,952 (62.8%) counties were excluded, including 1,937 due to CDC mortality data suppression (deaths < 10) and 15 due to missing covariate data. The final analytical sample comprised 1,156 counties.

Despite the geographic reduction, the representativeness analysis (Table 1) confirmed that the included counties encompassed 88.6% of the total US population (294,868,789 residents). Statistical comparisons revealed significant sociodemographic disparities between the included and excluded samples. Excluded counties were predominantly characterized by a complete lack of resident dermatologists (median density: 0.00 vs. 1.97, *P* < 0.01), older populations (median age: 43.00 vs. 40.55, *P* < 0.01), and a higher proportion of mobile homes (median percentile: 0.79 vs. 0.52, *P* < 0.01). This indicates that our analytical sample robustly captures melanoma mortality dynamics in moderately to densely populated regions.

**Table 1:** Baseline characteristics and representativeness analysis of counties in the contiguous United States, 2018–2024.

Variable	Included (*N* = 1,156)	Excluded (*N* = 1,952)	*P*-value
Total population coverage	294,868,789 (88.6%)	37,877,562 (11.4%)	—
medn_age	40.55 (37.77–43.60)	43.00 (40.30–46.00)	< 0.01
Res_Derm_Density	1.97 (0.00–4.02)	0.00 (0.00–0.00)	< 0.01
Env_UV_Exposure	4387.24 (4012.76–4748.08)	4507.43 (4211.87–4804.42)	< 0.01
EPL_MINRTY	0.25 (0.10–0.42)	0.23 (0.11–0.50)	< 0.01
EPL_POV200	0.48 (0.27–0.61)	0.62 (0.50–0.74)	< 0.01
EPL_NOHSDP	0.45 (0.31–0.56)	0.59 (0.43–0.73)	< 0.01
EPL_UNEMP	0.43 (0.29–0.52)	0.43 (0.28–0.56)	< 0.01
EPL_RENTER	0.38 (0.25–0.47)	0.38 (0.31–0.46)	< 0.01
EPL_HOUBDN	0.39 (0.25–0.48)	0.34 (0.25–0.44)	< 0.01
EPL_UNINSUR	0.42 (0.27–0.59)	0.57 (0.42–0.73)	< 0.01
EPL_NOINT	0.52 (0.29–0.65)	0.75 (0.64–0.85)	< 0.01
EPL_AGE65	0.54 (0.39–0.65)	0.67 (0.56–0.78)	< 0.01
EPL_AGE17	0.46 (0.33–0.56)	0.52 (0.40–0.61)	< 0.01
EPL_DISABL	0.54 (0.32–0.68)	0.70 (0.53–0.82)	< 0.01
EPL_LIMENG	0.26 (0.13–0.40)	0.24 (0.12–0.40)	0.42
EPL_MOBILE	0.52 (0.19–0.72)	0.79 (0.68–0.88)	< 0.01
EPL_GROUPQ	0.42 (0.29–0.52)	0.52 (0.38–0.69)	< 0.01
EPL_OZONE	0.24 (0.00–0.54)	0.00 (0.00–0.32)	< 0.01
EPL_PM	0.00 (0.00–0.50)	0.00 (0.00–0.59)	< 0.05
EPL_DSLPM	0.30 (0.15–0.45)	0.11 (0.04–0.19)	< 0.01
EPL_TOTCR	0.05 (0.04–0.45)	0.04 (0.02–0.33)	< 0.01
EPL_NPL	0.02 (0.00–0.12)	0.00 (0.00–0.00)	< 0.01
EPL_TRI	0.41 (0.32–0.51)	0.30 (0.16–0.39)	< 0.01
EPL_TSD	0.27 (0.15–0.42)	0.04 (0.00–0.23)	< 0.01
EPL_RMP	0.29 (0.17–0.43)	0.34 (0.00–0.67)	< 0.01
EPL_COAL	0.00 (0.00–0.00)	0.00 (0.00–0.00)	0.53
EPL_LEAD	0.00 (0.00–0.00)	0.00 (0.00–0.00)	0.43
EPL_PARK	0.71 (0.63–0.79)	0.85 (0.77–0.91)	< 0.01
EPL_HOUAGE	0.38 (0.18–0.53)	0.49 (0.38–0.63)	< 0.01
EPL_WLKIND	0.69 (0.53–0.77)	0.81 (0.74–0.87)	< 0.01
EPL_RAIL	0.43 (0.32–0.53)	0.39 (0.18–0.49)	< 0.01
EPL_ROAD	0.34 (0.25–0.43)	0.20 (0.13–0.27)	< 0.01
EPL_AIRPRT	0.18 (0.11–0.29)	0.36 (0.17–0.59)	< 0.01
EPL_IMPWTR	0.42 (0.27–0.62)	0.27 (0.18–0.39)	< 0.01
EPL_ASTHMA	0.53 (0.31–0.68)	0.58 (0.39–0.73)	< 0.01
EPL_CANCER	0.59 (0.41–0.72)	0.71 (0.57–0.82)	< 0.01
EPL_CHD	0.58 (0.34–0.75)	0.80 (0.69–0.89)	< 0.01
EPL_MHLTH	0.49 (0.31–0.63)	0.55 (0.33–0.70)	< 0.01
EPL_DIABETES	0.49 (0.27–0.65)	0.70 (0.54–0.84)	< 0.01
EPL_NEHD	0.41 (0.20–0.70)	0.46 (0.16–0.72)	0.86
EPL_BURN	0.00 (0.00–0.00)	0.00 (0.00–0.01)	< 0.01
EPL_SMOKE	0.53 (0.23–0.77)	0.72 (0.38–0.94)	< 0.01
EPL_CFLD	0.00 (0.00–0.00)	0.00 (0.00–0.00)	< 0.01
EPL_DRGT	0.35 (0.06–0.64)	0.49 (0.28–0.76)	< 0.01
EPL_HRCN	0.34 (0.02–0.57)	0.11 (0.00–0.46)	< 0.01
EPL_RFLD	0.33 (0.16–0.52)	0.16 (0.05–0.34)	< 0.01
EPL_SWND	0.55 (0.32–0.72)	0.57 (0.36–0.67)	0.83
EPL_TRND	0.08 (0.01–0.23)	0.50 (0.23–0.78)	< 0.01

Data are presented as median (interquartile range). The analytical sample (*N* = 1,156) represents 88.6% of the contiguous US population. Excluded counties (*N* = 1,952) comprised 1,937 counties with suppressed melanoma mortality data and 15 counties with missing covariate data. *P*-values were calculated using the two-sided Mann–Whitney *U* test.

### 3.2 Spatial distribution of melanoma mortality

**Figure 2:**
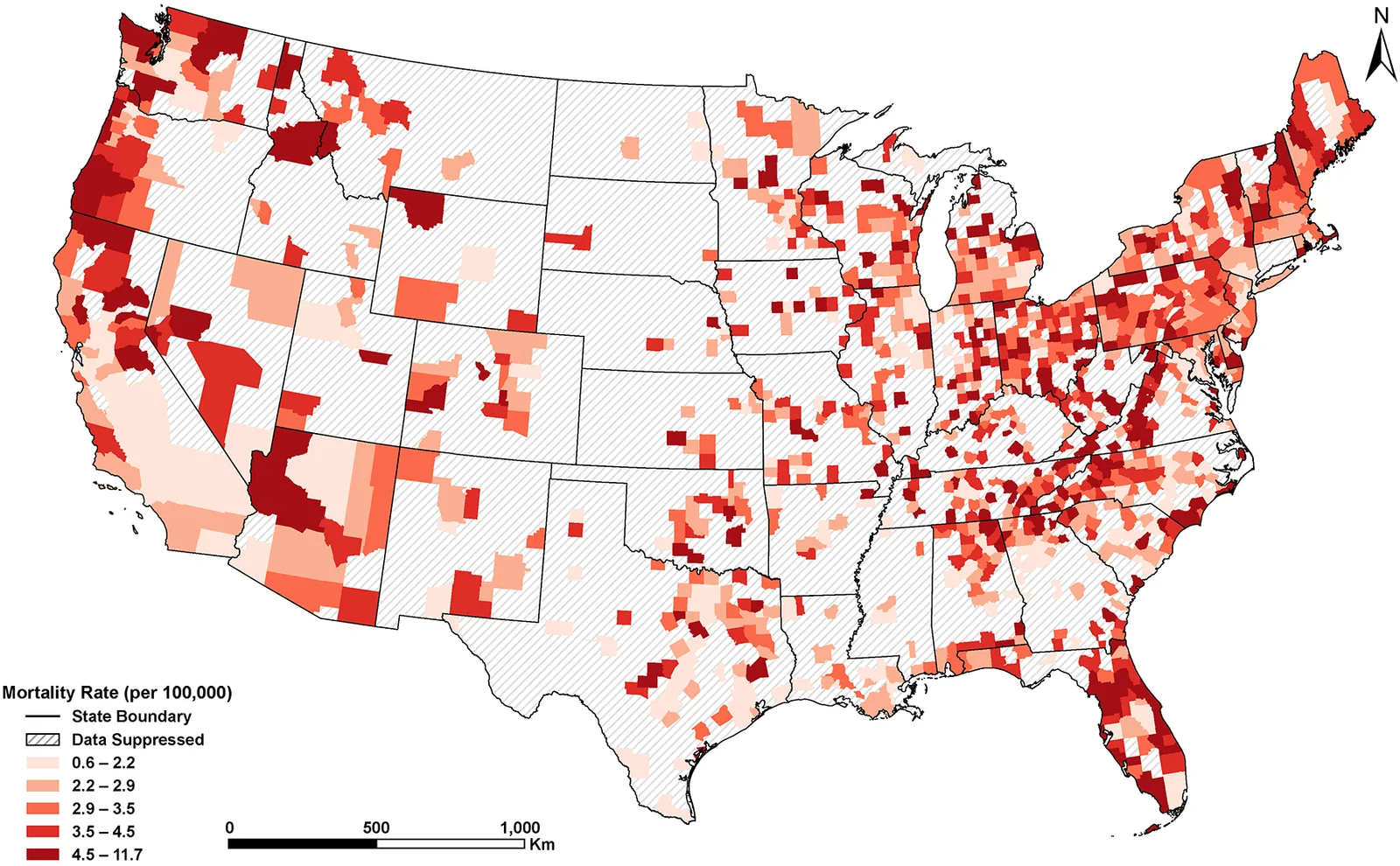
Spatial distribution of the average annual melanoma crude mortality rate across the contiguous United States (2018–2024).

The average annual melanoma crude mortality rate (2018–2024) exhibited a non-random, highly clustered spatial distribution across the contiguous US (Figure 2). Visualizing the mortality rates by quintiles revealed that counties in the highest mortality tier (4.5–11.7 per 100,000) were distinctly concentrated in the Southeastern states, the Appalachian region, and select areas of the Pacific Northwest. Conversely, lower mortality rates were more frequently observed in the Southwestern and South-Central regions. The presence of these stark geographical disparities empirically justified the necessity of employing spatial regression techniques.

### 3.3 Machine learning-based feature selection

Prior to regression modeling, severe multicollinearity among the initial EJI indicators was mitigated via iterative VIF screening, which excluded highly redundant variables (e.g., renter proportion, VIF = 88,528.72; Supplementary Table S2).

**Figure 3:**
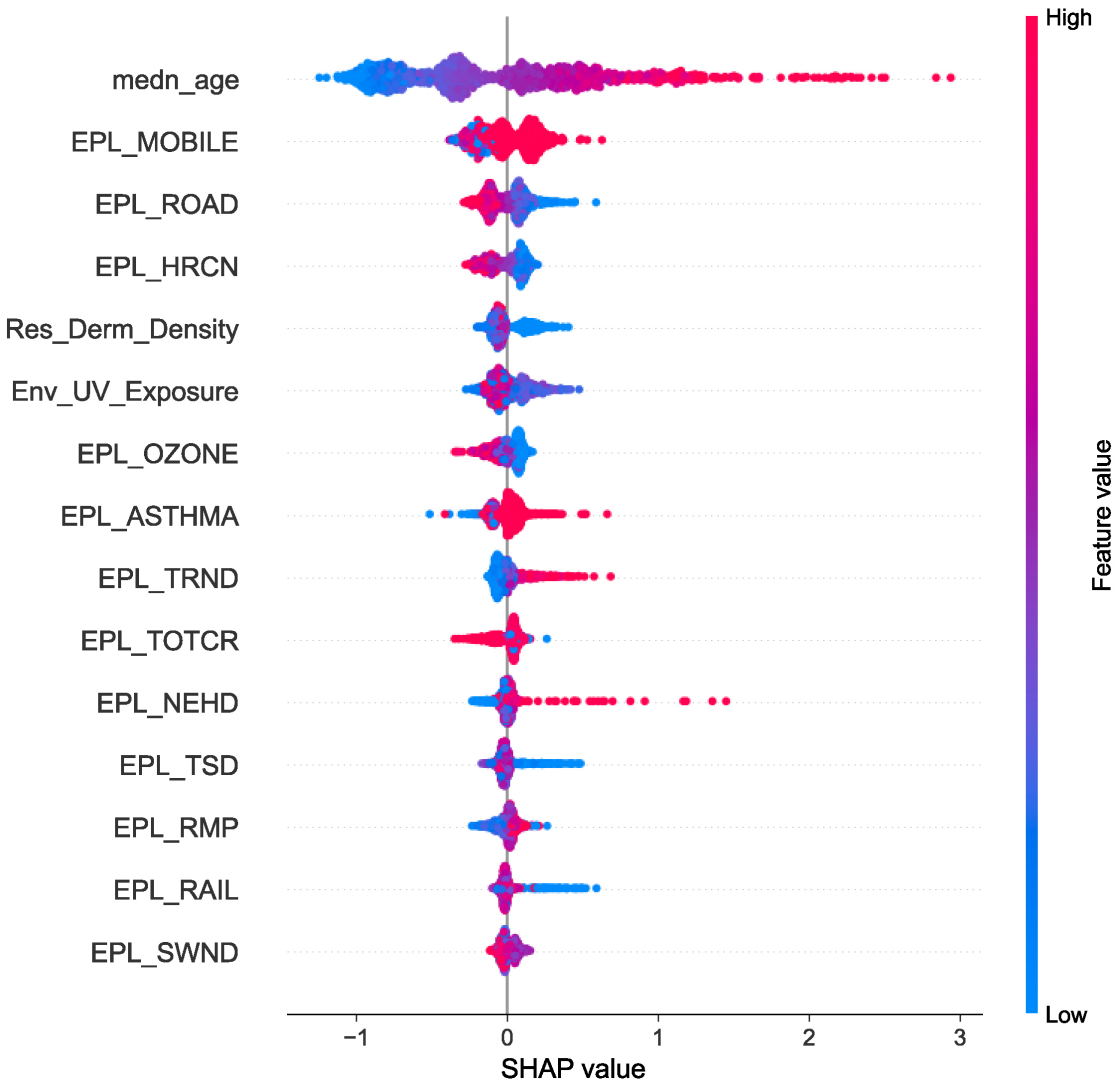
Global feature importance and impact of environmental and sociodemographic predictors on melanoma mortality based on the XGBoost-SHAP framework. Predictors are ranked along the y-axis based on their global importance (mean absolute SHAP value). Each point represents a single US county. The *x*-axis indicates the SHAP value, where positive values signify an increased risk of melanoma mortality and negative values signify a decreased risk. The color gradient represents the feature value, ranging from low (blue) to high (red).

The global importance of the retained features was subsequently evaluated using the XGBoost-SHAP framework (Figure 3). To ensure the robustness of these findings, a five-fold cross validation was performed, and the results are presented alongside the global rankings in Table 2. Median age emerged as the most dominant predictor (mean |SHAP| = 0.5958), followed by the proportion of mobile homes (0.1488) and high-volume road proximity (0.1071). The cross validation confirmed that the hierarchy of the top predictors remained highly stable across independent test sets. Although a minor rank permutation was observed between ozone exposure and asthma prevalence during cross validation, their mean SHAP values were statistically equivalent as the difference was well within their respective standard deviation margins. During the stepwise AICc optimization (Table 2), candidates for inclusion were derived from the top ten highest-ranked variables in the SHAP analysis and were sequentially introduced to the base model. Notably, while asthma prevalence and total cancer risk ranked high in non-linear SHAP importance, they failed to improve model parsimony and were excluded. The final optimal combination comprised 8 predefined and selected predictors (Final Model AICc = 3,248.38).

**Table 2:** Variable selection process combining XGBoost-derived global SHAP importance, 5-fold cross-validated SHAP stability, and stepwise AICc optimization.

Global rank	Variable	Global mean |SHAP|	Cross-validated mean |SHAP| ±SD	Selection status	Cumulative AICc
1	medn_age_20	0.5958	0.5903 ± 0.0370	Predefined core covariate	3465.21
2	EPL_MOBILE	0.1488	0.1376 ± 0.0140	Stepwise inclusion	3459.82
3	EPL_ROAD	0.1071	0.1123 ± 0.0170	Stepwise inclusion	3354.25
4	EPL_HRCN	0.1022	0.1058 ± 0.0084	Stepwise inclusion	3301.21
5	Res_Derm_Density	0.0901	0.0832 ± 0.0115	Predefined core covariate	—
6	Env_UV_Exposure	0.0895	0.0811 ± 0.0078	Predefined core covariate	—
7	EPL_OZONE	0.0701	0.0654 ± 0.0198	Stepwise inclusion	3272.87
8	EPL_ASTHMA	0.0682	0.0677 ± 0.0120	Excluded	—
9	EPL_TRND	0.0663	0.0639 ± 0.0105	Stepwise inclusion	3248.38
10	EPL_TOTCR	0.0654	0.0601 ± 0.0103	Excluded	—

Variables with VIF greater than 10 were excluded prior to feature selection. The global mean |SHAP| values dictate the primary rank sequence used for the stepwise linear evaluation. The cross-validated SHAP column demonstrates the stability of this ranking across five independent out-of-fold test sets. Minor rank permutations within the margin of error confirm the robustness of the non-linear feature extraction.

### 3.4 Model performance and spatial diagnostics

The performance diagnostics of the Global OLS, traditional GWR, and MGWR models are summarized in Table 3. The MGWR model demonstrated superior explanatory power, achieving the highest goodness-of-fit (*R*^2^ = 0.6664, Adjusted *R*^2^ = 0.6326) and the lowest AICc value (2,247.84) compared to OLS (AICc = 2,386.37) and GWR (AICc = 2,320.41). Furthermore, residual spatial autocorrelation, which was significantly positive in the OLS model (Moran's *I* = 0.1497, *P* < 0.01), was substantially reduced after MGWR calibration (Moran's *I* = −0.0575, *P* < 0.01). Although statistically significant, the residual Moran's *I* was close to zero, indicating only weak residual spatial dependence and an approximately random spatial distribution.

**Table 3:** Performance comparison of global (OLS), local (GWR), and multiscale (MGWR) regression models.

Model	*R* ^2^	Adjusted *R*^2^	AICc	RSS	Residual Moran's *I*	*P*-value
OLS	0.5458	0.5426	2386.37	525.04	0.1497	< 0.01
GWR	0.6531	0.6139	2320.41	401.05	0.0275	< 0.05
MGWR	0.6664	0.6326	2247.84	385.65	−0.0575	< 0.01

### 3.5 Spatially varying effects and scale differences

**Figure 4:**
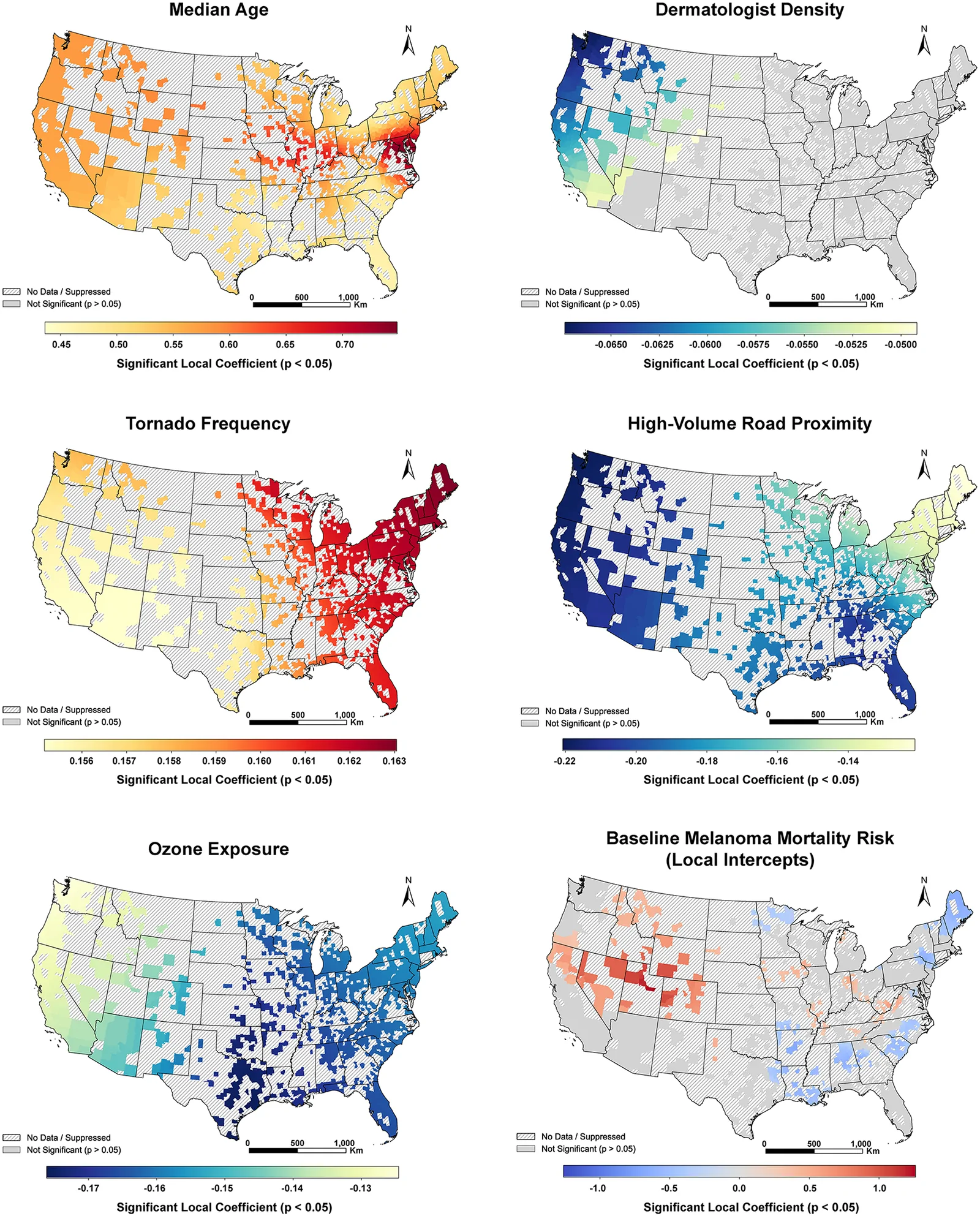
Spatially varying coefficients of local baseline risk and significant environmental and sociodemographic predictors for melanoma mortality in the contiguous United States (2018–2024). Maps display local regression coefficients (β) from the MGWR model. Six key components are visualized; three global-scale predictors (UV Exposure, Hurricane Frequency, and Mobile Homes) are omitted due to universal non-significance (*P* ≥ 0.05) and are provided in Supplementary Figure S1. Red/warm tones indicate positive associations (increased risk); blue/cool tones indicate negative associations (protective effect). Solid gray represents areas where the local relationship is not statistically significant (*P* ≥ 0.05).

Table 4 details the optimal bandwidths, and Figure 4 visualizes the spatially varying local coefficients derived from the MGWR model. The spatial scales of the predictors exhibited substantial variation. The baseline mortality risk, represented by the local intercept, operated at a highly localized scale with a bandwidth of 38. Significant positive baseline coefficients were clustered heavily in the Mountain West and Midwest regions, such as Utah, Colorado, and Wyoming, whereas negative baseline risks were primarily concentrated along the East Coast and Southeast. The association between median age and melanoma mortality was identified at a regional scale with a bandwidth of 233, showing a universally significant positive association nationwide that was most pronounced (coefficients up to 0.7485) in the Mid-Atlantic and Northeast regions. Similarly, high-volume road proximity operated at a broad regional scale with a bandwidth of 829, showing a widespread negative association characterized by negative coefficients that were strongest in the Western and Southwestern United States.

**Table 4:** . Statistical summary of local coefficients and optimal bandwidths from the MGWR model.

Predictor	Optimal bandwidth	Spatial scale	Min	Mean	Max
Intercept	38	Local	−0.6677	−0.0057	1.2615
medn_age_20	233	Regional	0.4363	0.5556	0.7485
Res_Derm_Density	1,115	Global	−0.0674	−0.0278	−0.0166
Env_UV_Exposure	1,155	Global	−0.1126	−0.1073	−0.0894
EPL_MOBILE	1,155	Global	0.0215	0.0259	0.0341
EPL_ROAD	829	Regional	−0.2208	−0.177	−0.1212
EPL_HRCN	1,155	Global	−0.0523	−0.0363	−0.0302
EPL_OZONE	1,120	Global	−0.1761	−0.1598	−0.1245
EPL_TRND	1,155	Global	0.1552	0.1599	0.163

The spatial scale for each predictor was categorized based on the ratio of its optimal bandwidth to the total number of observations (*N* = 1,156): local (bandwidth < 10% of *N*, i.e., < 116), regional (10%−75% of *N*, i.e., 116–867), and global (>75% of *N*, i.e., >867).

Predictors including dermatologist density, ozone exposure, and tornado frequency operated at global scales with bandwidths exceeding 1,100, indicating that their spatial variation remained relatively smooth across the country. Dermatologist density demonstrated a widespread inverse association with melanoma mortality (mean coefficient = −0.0278) that reached statistical significance primarily in the Pacific Northwest. Ozone exposure also showed a significant negative association, which was predominantly observed in the Midwest and South. Conversely, tornado frequency exhibited a significant positive association with melanoma mortality, with significant associations particularly concentrated in the Northeast and Midwest regions.

Several other global-scale variables, specifically UV exposure, hurricane frequency, and the proportion of mobile homes, were retained in the final model with maximum bandwidths of 1,155. However, their localized marginal associations failed to reach statistical significance (*P* ≥ 0.05) in any of the 1,156 analyzed counties. The spatially stable but non-significant coefficient distributions for these three predictors are documented in Supplementary Figure S1.

To evaluate whether the observed protective associations of built-environment indicators were influenced by underlying urbanization differences, we performed an additional sensitivity analysis incorporating a binary urban-rural classification derived from the USDA Rural-Urban Continuum Codes (RUCC 2023). After adjustment for metropolitan status, the protective associations of high-volume road proximity and ozone exposure remained consistent, although the effect magnitudes were attenuated. In the MGWR sensitivity model, the mean coefficient of EPL_ROAD changed from −0.1770 to −0.1593, while EPL_OZONE changed from −0.1598 to −0.1294 (Supplementary Table S3). Similarly, global OLS sensitivity analyses demonstrated statistically significant negative associations after urbanicity adjustment (EPL_ROAD: β = −0.1393, *P* < 0.001; EPL_OZONE: β = −0.0866, *P* = 0.0001). These findings indicate that urban-rural differences partially contributed to, but did not fully explain, the observed protective associations of road proximity and ozone exposure.

## 4 Discussion

### 4.1 Superiority of the multiscale spatial framework

This study represents a comprehensive national-level investigation into the spatial determinants of melanoma mortality in the contiguous United States (2018–2024), utilizing the latest EJI and MGWR framework. Our findings empirically demonstrate that factors associated with melanoma mortality operate across heterogeneous spatial scales rather than following a uniform spatial pattern. The MGWR model significantly outperformed global OLS models by substantially reducing residual spatial autocorrelation and uncovering variable-specific spatial scales, ranging from localized baseline risks to global-scale protective effects associated with healthcare access. Importantly, the significant spatially varying intercept indicates that residual regional heterogeneity remained after accounting for the measured predictors, suggesting the potential influence of unmeasured socioeconomic, healthcare, behavioral, or environmental factors.

### 4.2 The overriding threat of population aging

Consistent with the biological natural history of cutaneous melanoma, population aging (medn_age) emerged as the most dominant predictor of mortality in the XGBoost-SHAP analysis, exerting a robust regional-scale impact. Older populations inherently carry a higher cumulative burden of uncorrected somatic mutations from lifelong environmental exposures, compounded by age-related immunosenescence which impairs tumor surveillance (25, 26). This finding remained consistent despite potential demographic selection bias introduced by the analytical sample restriction. Specifically, as indicated by our representativeness analysis (Table 1), the excluded rural counties were older but sparsely populated and were systematically removed due to CDC WONDER privacy suppression (< 10 deaths) (27–29). The persistence of age as the dominant predictor despite the exclusion of these older counties further supports its robust association with melanoma mortality.

Furthermore, while median age was a statistically robust predictor, its epidemiological interpretation differs from proportion-based age metrics. In dermatological research, the proportion of residents aged 65 or older is generally considered more clinically relevant because it more directly captures the population subgroup at highest risk (16, 17). However, the EJI elderliness indicator (EPL_AGE65) was excluded during VIF screening due to severe multicollinearity with other social vulnerability indicators (Supplementary Table S2). Therefore, county-level median age from the AHRF dataset was retained as a practical indicator of overall population age structure. Although this approach effectively adjusted for population aging, it may not fully capture the specific contribution of the oldest age groups in regions with highly skewed age distributions, highlighting the need for future studies incorporating more granular age-distribution metrics.

Our local coefficient maps (Figure 4) revealed that the mortality risk associated with aging was particularly magnified in the Northeast and Mid-Atlantic regions. This regional amplification likely reflects the demographic reality of “aging-in-place” phenomena in these states, emphasizing that national cancer control strategies must prioritize geriatric dermatology screening in these specific geographical clusters.

### 4.3 Global protective effects of dermatologist density

A critical finding of our study is the global-scale inverse association of dermatologist density with melanoma mortality. The wide optimal bandwidth (>1,100) indicates that the association between dermatologist density and melanoma mortality operates at a broad national scale. Prior studies have established that counties with severe shortages of dermatologists suffer from delayed diagnoses, leading to thicker, later-stage melanomas at presentation (30). Our findings expand upon this by suggesting that higher specialist workforce availability is associated with lower melanoma mortality across the United States, contrasting sharply with the severe rural health disparities observed among vulnerable populations (31). The spatial mapping underscores that addressing the maldistribution of the oncology workforce may represent an important strategy for mitigating melanoma mortality disparities (32).

### 4.4 Deciphering the urban-environmental paradox

An intriguing, counter-intuitive observation in our results is the significant negative (protective) association between melanoma mortality and specific built-environment burdens, namely high-volume road proximity (EPL_ROAD) and ozone exposure (EPL_OZONE). From an ecotoxicological standpoint, traffic pollution and ozone are detrimental. However, in spatial epidemiology, these variables frequently act as robust proxies for urbanization and metropolitan status (33, 34).

Counties with high road density and elevated ozone are predominantly urban centers that house premier NCI-designated cancer centers and may offer greater availability of genomic tumor testing and immunotherapy services (35). Our sensitivity analysis incorporating USDA RUCC-based urban-rural classification further evaluated whether these associations were attributable solely to metropolitan status. Although adjustment for metropolitan status attenuated the protective associations of both EPL_ROAD and EPL_OZONE, the associations remained directionally consistent and statistically significant, suggesting that urbanicity contributes to but does not fully account for these observed relationships. This residual association may reflect broader differences in healthcare accessibility and socioeconomic conditions between metropolitan and rural communities, factors that have been associated with delayed melanoma diagnosis and more advanced disease presentation (36). Conversely, rural residents in areas with pristine air quality often experience fatal treatment delays potentially related to geographic isolation.

### 4.5 Climate hazards and social deprivation

This study provides novel evidence demonstrating an association between climate vulnerability and melanoma mortality. Tornado frequency (EPL_TRND) operated at a global scale and exhibited a significant positive association with melanoma mortality, particularly in the Midwest. While natural disasters do not directly cause melanoma, they may disrupt healthcare infrastructure and are potentially associated with fragmented oncology care, delayed adjuvant therapies, and missed screenings (37, 38). Recent scoping reviews confirm that older adults with cancer face disproportionate mortality risks following climate-amplified disasters due to prolonged systemic disruptions (39).

Furthermore, social vulnerability, heavily driven by extreme poverty proxies such as the proportion of mobile homes (EPL_MOBILE), was highly ranked in our SHAP analysis. Neighborhood deprivation is an independent risk factor for thicker melanoma at diagnosis, as marginalized populations frequently lack adequate health insurance or health literacy to seek timely surgical intervention (40, 41).

### 4.6 The “UV paradox”: incidence vs. mortality decoupling

Interestingly, while environmental UV exposure (Env_UV_Exposure) is the universally acknowledged primary carcinogen for melanoma incidence, its localized marginal impact on melanoma *mortality* failed to reach statistical significance (*P* ≥ 0.05) in our MGWR analysis.

This apparent paradox perfectly aligns with recent paradigm shifts in dermatological epidemiology. Adewole et al. (34) demonstrated a profound decoupling between melanoma incidence and mortality in the US: while incidence correlates strongly with UV radiation and diagnostic scrutiny (e.g., skin biopsies of indolent lesions), mortality is predominantly associated with late-stage presentation and socioeconomic deprivation rather than ambient sunlight (34, 42). Once our model adjusted for age and healthcare access, the residual explanatory power of UV exposure on the ultimate fatal outcome became statistically negligible. This underscores that public health campaigns must transcend mere sun-avoidance messaging and address the socioeconomic barriers to early tumor detection (42).

### 4.7 Strengths and limitations

The primary strength of this study is the integration of the novel EJI 2024 database with explainable artificial intelligence (SHAP) and multiscale spatial regression, enabling the assessment of complex environmental and social determinants while accounting for multicollinearity and spatial non-stationarity. However, several limitations should be acknowledged.

First, as an ecological study, our findings are subject to ecological fallacy, and county-level associations cannot be directly translated into individual-level clinical outcomes. For example, the observed protective associations of traffic proximity and ozone may reflect broader county-level healthcare advantages rather than individual biological effects. Aggregated geographic data may also obscure finer-scale spatial heterogeneity due to the modifiable areal unit problem (MAUP) (43, 44).

Second, CDC WONDER privacy suppression resulted in the exclusion of sparsely populated rural counties. Although our analysis covered 88.6% of the US population, underrepresentation of hyper-rural regions may limit the characterization of areas with extreme healthcare deprivation. Furthermore, although adaptive kernels improve estimation stability by maintaining sufficient neighboring observations, spatial sparsity may require substantially larger bandwidths in rural regions, potentially reducing spatial resolution and resulting in local coefficients that reflect broader regional trends rather than highly localized county-level patterns. Therefore, spatially heterogeneous effects in sparsely represented regions should be interpreted cautiously.

Third, because directly age-standardized mortality rates were unavailable at the required geographic resolution, crude mortality rates adjusted for median age were used. Although this approach accounts for overall demographic aging, it cannot fully replace direct age standardization, and residual confounding related to age structure may remain.

Finally, our analytical framework used tree-based machine learning for variable selection followed by linear MGWR modeling. Consequently, some potentially important non-linear relationships and interaction effects identified by SHAP could not be fully incorporated into the final additive spatial model, representing an inherent methodological trade-off in current spatial epidemiological modeling. The cross-sectional design also precludes causal inference between environmental exposures and melanoma mortality.

## 5 Conclusion

County-level melanoma mortality in the United States exhibited complex spatial heterogeneity associated with demographic, socioeconomic, healthcare, and environmental factors. Population aging and dermatologist availability showed the strongest associations with mortality patterns. The persistence of inverse associations observed for high-volume road proximity and ozone exposure after urbanicity adjustment suggests that these relationships may reflect broader differences in healthcare accessibility and socioeconomic conditions rather than direct protective environmental effects. Climate-related vulnerability, including tornado frequency, was also positively associated with melanoma mortality, suggesting a potential role of healthcare disruption and related disparities. These findings support the value of spatially targeted public health strategies and resource allocation approaches, particularly for aging, disaster-prone, and socioeconomically vulnerable rural communities.

## Data Availability

The original contributions presented in the study are included in the article/Supplementary material, further inquiries can be directed to the corresponding authors.
