# Supplementary material for: A multiscale geospatial analysis integrating explainable machine learning to characterize spatial disparities in melanoma mortality associated with environmental justice and healthcare access in the contiguous United States
Source: Front Public Health. 2026 Aug 20;14:1866117. doi: 10.3389/fpubh.2026.1866117 (PMC13538888; doi:10.3389/fpubh.2026.1866117)
Supplement: Supplementary file 1 [file Supplementary_File_1.docx]

**Table S1. Comprehensive data dictionary of all candidate variables considered in the study.**

The prefix EPL_ denotes the Estimated Percentile rank (ranging from 0 to 1) of the respective indicator at the county level. All EJI percentile variables indicate that a higher value corresponds to a higher level of environmental, social, or health burden.

| **Variable Code** | **Domain** | **Variable Description** |
| --- | --- | --- |
| **Predefined Core Covariates** |  |  |
| medn_age | Demographics | Median age of the county population. |
| Res_Derm_Density | Healthcare Access | Density of resident, non-federal dermatologists per 100,000 population. |
| Env_UV_Exposure | Environmental | Daily mean ultraviolet (UV) irradiance (Wh/m²) averaged. |
| **EJI: Social Vulnerability Module** | *(Percentile Ranks)* |  |
| EPL_MINRTY | Demographics | Percentile rank of minority population proportion. |
| EPL_POV200 | Socioeconomic | Percentile rank of persons living below 200% of the federal poverty level. |
| EPL_NOHSDP | Socioeconomic | Percentile rank of persons (age 25+) with no high school diploma. |
| EPL_UNEMP | Socioeconomic | Percentile rank of unemployed civilian population (age 16+). |
| EPL_RENTER | Housing | Percentile rank of renter-occupied housing units proportion. |
| EPL_HOUBDN | Housing | Percentile rank of households severely burdened by housing costs (>30% of income). |
| EPL_UNINSUR | Socioeconomic | Percentile rank of uninsured persons proportion. |
| EPL_NOINT | Infrastructure | Percentile rank of households without a broadband internet subscription. |
| EPL_AGE65 | Demographics | Percentile rank of persons aged 65 and older. |
| EPL_AGE17 | Demographics | Percentile rank of persons aged 17 and younger. |
| EPL_DISABL | Health/Social | Percentile rank of civilian noninstitutionalized population with a disability. |
| EPL_LIMENG | Demographics | Percentile rank of persons with limited English proficiency. |
| EPL_MOBILE | Housing | Percentile rank of housing units designated as mobile homes. |
| EPL_GROUPQ | Housing | Percentile rank of persons living in group quarters (e.g., nursing homes, prisons). |
| **EJI: Environmental Burden Module** | *(Percentile Ranks)* |  |
| EPL_OZONE | Air Pollution | Percentile rank of annual mean days above the ozone regulatory standard. |
| EPL_PM | Air Pollution | Percentile rank of annual mean PM2.5 concentrations. |
| EPL_DSLPM | Air Pollution | Percentile rank of diesel particulate matter ambient concentrations. |
| EPL_TOTCR | Toxics | Percentile rank of estimated lifetime air toxics cancer risk. |
| EPL_NPL | Built Environment | Percentile rank of tract's area within a 1-mile buffer of National Priorities List (Superfund) sites. |
| EPL_TRI | Built Environment | Percentile rank of area within a 1-mile buffer of Toxic Release Inventory facilities. |
| EPL_TSD | Built Environment | Percentile rank of area within a 1-mile buffer of Treatment, Storage, and Disposal facilities. |
| EPL_RMP | Built Environment | Percentile rank of area within a 1-mile buffer of Risk Management Plan (hazardous chemical) sites. |
| EPL_COAL | Built Environment | Percentile rank of area within a 1-mile buffer of coal mines. |
| EPL_LEAD | Housing Quality | Percentile rank of housing built before 1960 (proxy for potential lead paint exposure). |
| EPL_PARK | Built Environment | Percentile rank of lack of access to recreational parks. |
| EPL_HOUAGE | Housing Quality | Percentile rank of housing built before 1980. |
| EPL_WLKIND | Built Environment | Percentile rank of low National Walkability Index score. |
| EPL_RAIL | Built Environment | Percentile rank of area within a 1-mile buffer of active railways. |
| EPL_ROAD | Built Environment | Percentile rank of area within a 1-mile buffer of high-volume roads or highways. |
| EPL_AIRPRT | Built Environment | Percentile rank of area within a 1-mile buffer of airports. |
| EPL_IMPWTR | Water Quality | Percentile rank of area within a 1-mile buffer of impaired surface water bodies. |
| **EJI: Health Vulnerability Module** | *(Percentile Ranks)* |  |
| EPL_ASTHMA | Chronic Disease | Percentile rank of the estimated prevalence of asthma among adults. |
| EPL_CANCER | Chronic Disease | Percentile rank of the estimated prevalence of cancer (excluding skin cancer) among adults. |
| EPL_CHD | Chronic Disease | Percentile rank of the estimated prevalence of coronary heart disease among adults. |
| EPL_MHLTH | Mental Health | Percentile rank of the estimated prevalence of poor mental health (≥14 days). |
| EPL_DIABETES | Chronic Disease | Percentile rank of the estimated prevalence of diagnosed diabetes among adults. |
| **EJI: Climate Vulnerability Module** | *(Percentile Ranks)* |  |
| EPL_NEHD | Climate/Weather | Percentile rank of the number of extreme heat days. |
| EPL_BURN | Climate/Weather | Percentile rank of area impacted by wildfires (burned areas). |
| EPL_SMOKE | Air Quality | Percentile rank of exposure to wildfire smoke plumes. |
| EPL_CFLD | Natural Disaster | Percentile rank of coastal flooding risk. |
| EPL_DRGT | Natural Disaster | Percentile rank of drought risk/exposure. |
| EPL_HRCN | Natural Disaster | Percentile rank of average annualized frequency of hurricane events. |
| EPL_RFLD | Natural Disaster | Percentile rank of riverine flooding risk. |
| EPL_SWND | Natural Disaster | Percentile rank of average annualized frequency of strong wind events. |
| EPL_TRND | Natural Disaster | Percentile rank of average annualized frequency of tornado events. |

**Table S2. Multicollinearity diagnostics for Environmental Justice Index (EJI) indicators: stepwise exclusion log and final VIF scores for retained variables.**

Multicollinearity was assessed using the Variance Inflation Factor (VIF). A stepwise exclusion procedure was implemented where the variable with the highest VIF was removed iteratively until all remaining indicators achieved a VIF < 10. High initial values reflect extreme redundancy among the original 45 candidate indicators.

| **Step** | **Variable** | **VIF** |
| --- | --- | --- |
| 1 | EPL_RENTER | 88528.72 |
| 2 | EPL_CHD | 66299.58 |
| 3 | EPL_NOINT | 43303.06 |
| 4 | EPL_MHLTH | 25368.57 |
| 5 | EPL_MINRTY | 22469.52 |
| 6 | EPL_HOUAGE | 20569.87 |
| 7 | EPL_DISABL | 16216.90 |
| 8 | EPL_GROUPQ | 14895.48 |
| 9 | EPL_AGE17 | 9856.52 |
| 10 | EPL_DIABETES | 8439.12 |
| 11 | EPL_CANCER | 4670.38 |
| 12 | EPL_AGE65 | 3631.65 |
| 13 | EPL_DSLPM | 2120.35 |
| 14 | EPL_HOUBDN | 77.35 |
| 15 | EPL_POV200 | 44.97 |
| 16 | EPL_LIMENG | 26.23 |
| 17 | EPL_UNINSUR | 13.39 |
| 18 | EPL_NOHSDP | 6.77 |
| 19 | EPL_ASTHMA | 6.65 |
| 20 | EPL_UNEMP | 6.45 |
| 21 | EPL_MOBILE | 5.81 |
| 22 | EPL_SMOKE | 4.93 |
| 23 | EPL_HRCN | 3.98 |
| 24 | EPL_PARK | 3.88 |
| 25 | EPL_WLKIND | 3.70 |
| 26 | EPL_ROAD | 3.20 |
| 27 | EPL_TRI | 2.68 |
| 28 | EPL_DRGT | 2.44 |
| 29 | EPL_TRND | 2.16 |
| 30 | EPL_RAIL | 2.12 |
| 31 | EPL_SWND | 2.06 |
| 32 | EPL_NEHD | 1.83 |
| 33 | EPL_TSD | 1.82 |
| 34 | EPL_PM | 1.72 |
| 35 | EPL_TOTCR | 1.72 |
| 36 | EPL_CFLD | 1.67 |
| 37 | EPL_IMPWTR | 1.53 |
| 38 | EPL_OZONE | 1.51 |
| 39 | EPL_BURN | 1.45 |
| 40 | EPL_RMP | 1.43 |
| 41 | EPL_AIRPRT | 1.26 |
| 42 | EPL_RFLD | 1.26 |
| 43 | EPL_COAL | 1.17 |
| 44 | EPL_NPL | 1.15 |
| 45 | EPL_LEAD | 1.10 |

**Table S3. Sensitivity analysis adjusting for urban-rural classification.**

| **Variable** | **Original MGWR β** | **Urban-adjusted MGWR β** | **Absolute change** |
| --- | --- | --- | --- |
| EPL_ROAD | -0.1770 | -0.1593 | 0.0177 |
| EPL_OZONE | -0.1598 | -0.1294 | 0.0303 |
| Is_Metro | — | -0.1941 | — |

**Figure S1. Spatially varying coefficients for predictors demonstrating global stationarity and universal non-significance in the MGWR model.**
